# Hemophagocytic Lymphohistiocytosis Associated With Disseminated Histoplasmosis in a Patient With Chronic Pancytopenia: A Rare Case

**DOI:** 10.7759/cureus.96726

**Published:** 2025-11-12

**Authors:** Gurjot Singh, Smitkumar Patel, Sandipkumar Patel, Didar Singh, Afshan Iqbal

**Affiliations:** 1 Internal Medicine, University of Connecticut, Waterbury, USA; 2 Internal Medicine, Gujarat Medical Education and Research Society Medical College and Hospital, Gandhinagar, IND; 3 Internal Medicine, Springfield Memorial Hospital, Springfield, USA; 4 Hospital Medicine, Springfield Clinic, Springfield, USA; 5 Internal Medicine/Hospital Medicine, Springfield Memorial Hospital, Southern Illinois University, Springfield, USA; 6 Internal Medicine/Hospital Medicine, Saint John Hospital, Southern Illinois University, Springfield, USA; 7 Infectious Disease, Springfield Clinic, Springfield, USA

**Keywords:** diffuse histoplasmosis, disseminated fungemia, hemophagocytic lymphohistiocytosis (hlh), secondary hemophagocytosis, sepsis

## Abstract

Hemophagocytic lymphohistiocytosis (HLH) is an uncommon yet life-threatening immune disorder in which dysregulated activity of cytotoxic T cells and macrophages leads to excessive cytokine release, uncontrolled inflammation, and damage to multiple organ systems. Secondary forms of HLH may develop in response to infections, malignancies, or autoimmune conditions. We present the case of an 80-year-old woman with longstanding pancytopenia who developed disseminated histoplasmosis complicated by HLH. This report highlights the diagnostic difficulties of HLH in older patients and stresses the importance of recognizing this syndrome promptly when opportunistic infections are suspected.

## Introduction

Hemophagocytic lymphohistiocytosis (HLH) is an uncommon but highly aggressive inflammatory syndrome that may develop in response to infections, autoimmune disorders, or malignancies. Although viral triggers are more frequently encountered, opportunistic fungal infections such as *Histoplasma capsulatum* can also precipitate HLH, particularly in immunocompromised or elderly individuals [1-3].

In this case, the patient satisfied several of the HLH-2004 diagnostic criteria. These included persistent fever, hepatosplenomegaly, cytopenias affecting multiple blood cell lines, markedly elevated ferritin levels, raised soluble interleukin-2 (IL-2) receptor (CD25), and bone marrow findings consistent with hemophagocytosis. A ferritin concentration greater than 500 µg/L is considered significant, and the markedly elevated levels in this patient provided strong diagnostic support. The increase in soluble IL-2 receptor further indicated excessive immune activation, while bone marrow examination revealed macrophages engulfing hematopoietic cells, a classic histopathological feature of HLH [2-5]. Taken together, the clinical picture and laboratory evidence strongly confirmed the diagnosis.

HLH represents a cytokine-driven hyperinflammatory state that often progresses to multi-organ dysfunction. Because its presentation can closely mimic severe sepsis, diagnosis is frequently delayed. Demonstrating hemophagocytosis on bone marrow biopsy remains an important supportive finding, though the broader clinical and laboratory context is equally critical [5-7]. Early recognition, initiation of antifungal therapy, and comprehensive supportive care are essential steps in improving outcomes for patients with HLH secondary to disseminated infections.

## Case presentation

**Figure 1:**
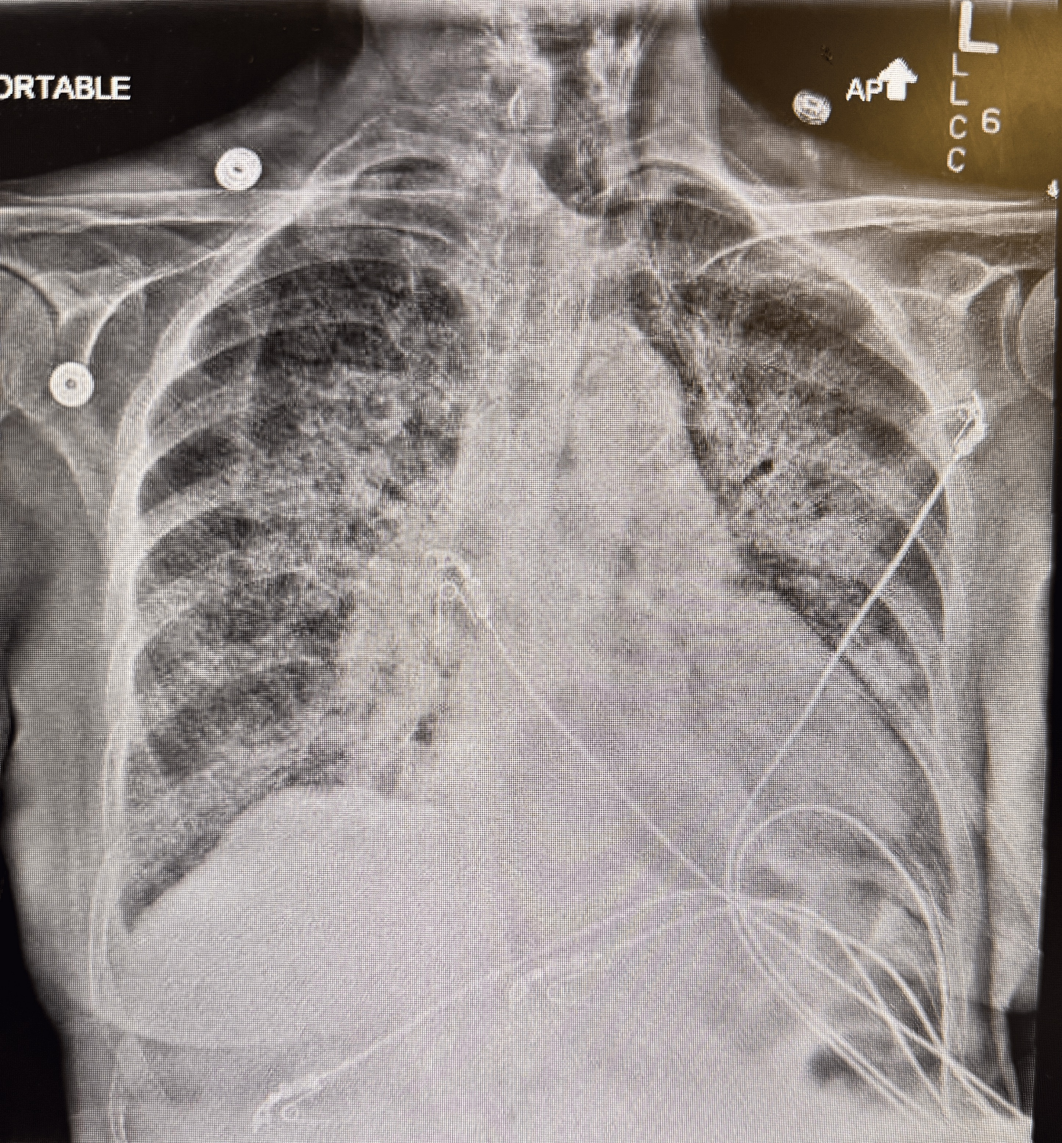
Chest X-ray showing bilateral multiple opacities.

An 80-year-old female with a longstanding history of chronic pancytopenia presented with a several-week history of fever, chills, and progressive dyspnea. Initial evaluation with chest radiography revealed patchy opacities in the right hilum and middle lobe, raising suspicion for community-acquired pneumonia (Figure 1). Within 24 hours of admission, the patient developed acute hypoxemic respiratory failure necessitating transfer to the intensive care unit (ICU) for supplemental oxygen support.

Laboratory investigations on admission confirmed pancytopenia, with hemoglobin at 8.7 g/L, platelets at 13 × 10⁹/L, and leukocytes at 0.4 × 10⁹/L, consistent with her chronic baseline, but notably lower than prior values. Abdominal imaging demonstrated hepatosplenomegaly. Additional laboratory abnormalities included markedly elevated serum ferritin (7,500 ng/mL), mildly increased aspartate aminotransferase (72 U/L), and hypoalbuminemia (24.0 g/L), indicating systemic inflammation and liver involvement (Table 1).

**Table 1:** Comparative laboratory values during hospitalization.

Parameters	Initial admission days	On discharge	Reference range
White blood cell count (1,000/mm³)	0.4	3.7	4–11
Platelet count (10⁹/µL)	13	20	150–450
Red blood cell count (million/µL)	2.49	8.2	4.0–5.1
Hemoglobin (g/dL)	8.7	8.2	12–14
Mean corpuscular volume (fL)	95	91	80–100
Mean corpuscular hemoglobin (pg)	35	33.5	27.5–33.2
Mean corpuscular hemoglobin concentration (g/dL)	34.7	35	33.4–35.5
Absolute neutrophil count (1,000/mm³)	0.2	2.2	2,500–7,000
Absolute lymphocyte count (1,000/mm³)	0.2	1.35	1,000–5,000
Serum iron (µg/dL)	22	125	40–170
Ferritin levels (ng/mL)	7,500	1,060	30–400
Vitamin B12 levels (pg/mL)	750	745	160–950
Reticulocyte count (%)	0.65	0.7	0.5–2.5%
Aspartate aminotransferase (U/L)	13	17	<40
Alanine aminotransferase (U/L)	21	23	<40
Lactate dehydrogenase (U/L)	109	103	<150
Creatinine (mg/dL)	1	0.9	<1.2
Triglyceride (mg/dL)	551	333	<150
Fibrinogen (mg/dL)	89	180	200–400

A comprehensive infectious workup was performed to identify potential triggers for hyperinflammatory states. Fungal testing was conducted due to the patient’s residence in an endemic region. The results revealed a negative 1,3-β-D-glucan (<31), but positive *Aspergillus galactomannan* (index 4.53) and urine *Histoplasma* antigen (>24), findings indicative of disseminated histoplasmosis. Viral serologies, including HIV, Epstein-Barr virus (EBV), cytomegalovirus (CMV), hepatitis B and C, and SARS-CoV-2, were all negative, effectively ruling out viral-driven HLH. Blood cultures and bacterial workup were negative, making bacterial sepsis less likely as a precipitant.

**Figure 2:**
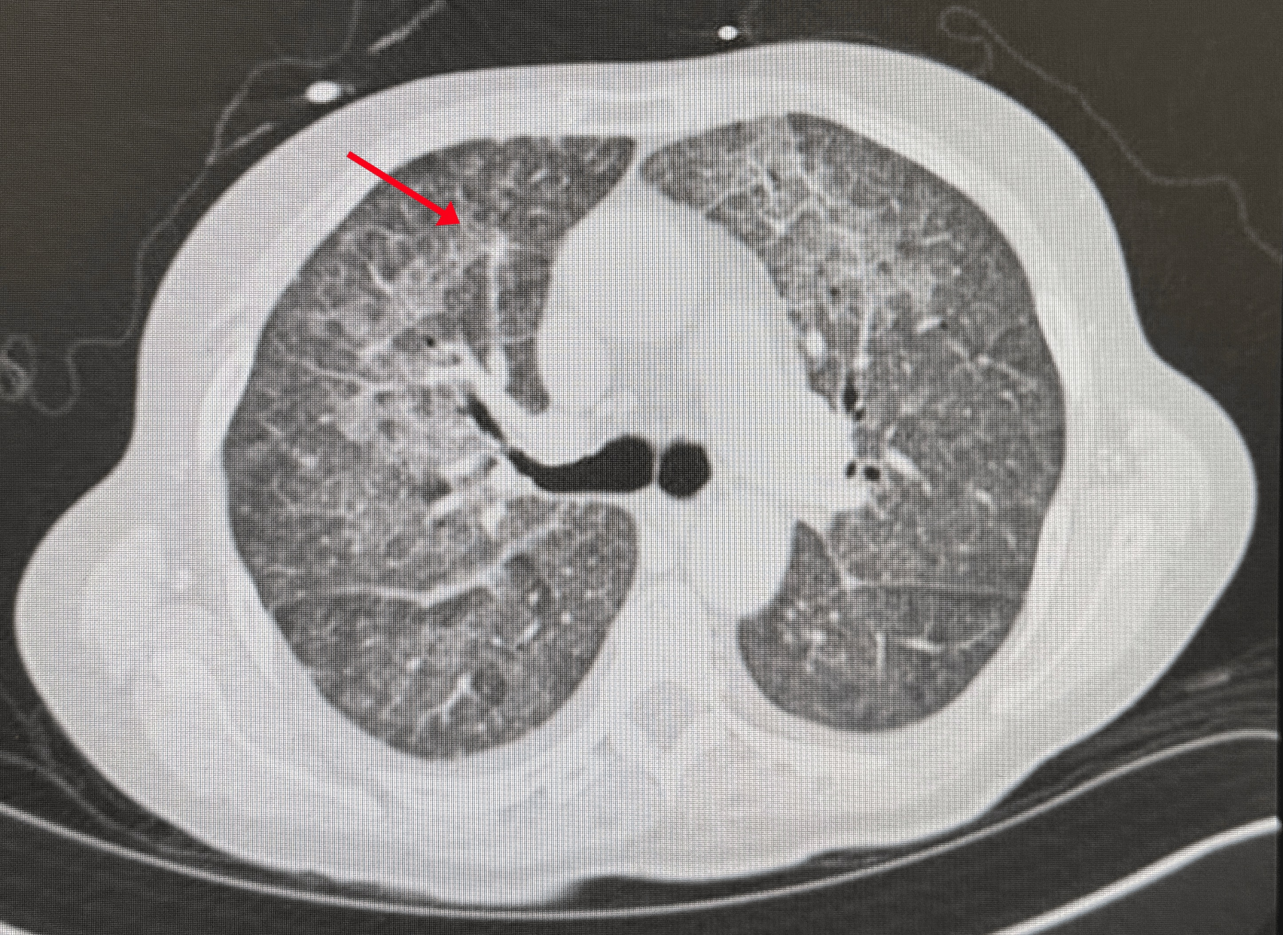
Chest CT revealing extensive ground-glass opacities with interlobular septal thickening (red arrow).

**Figure 3:**
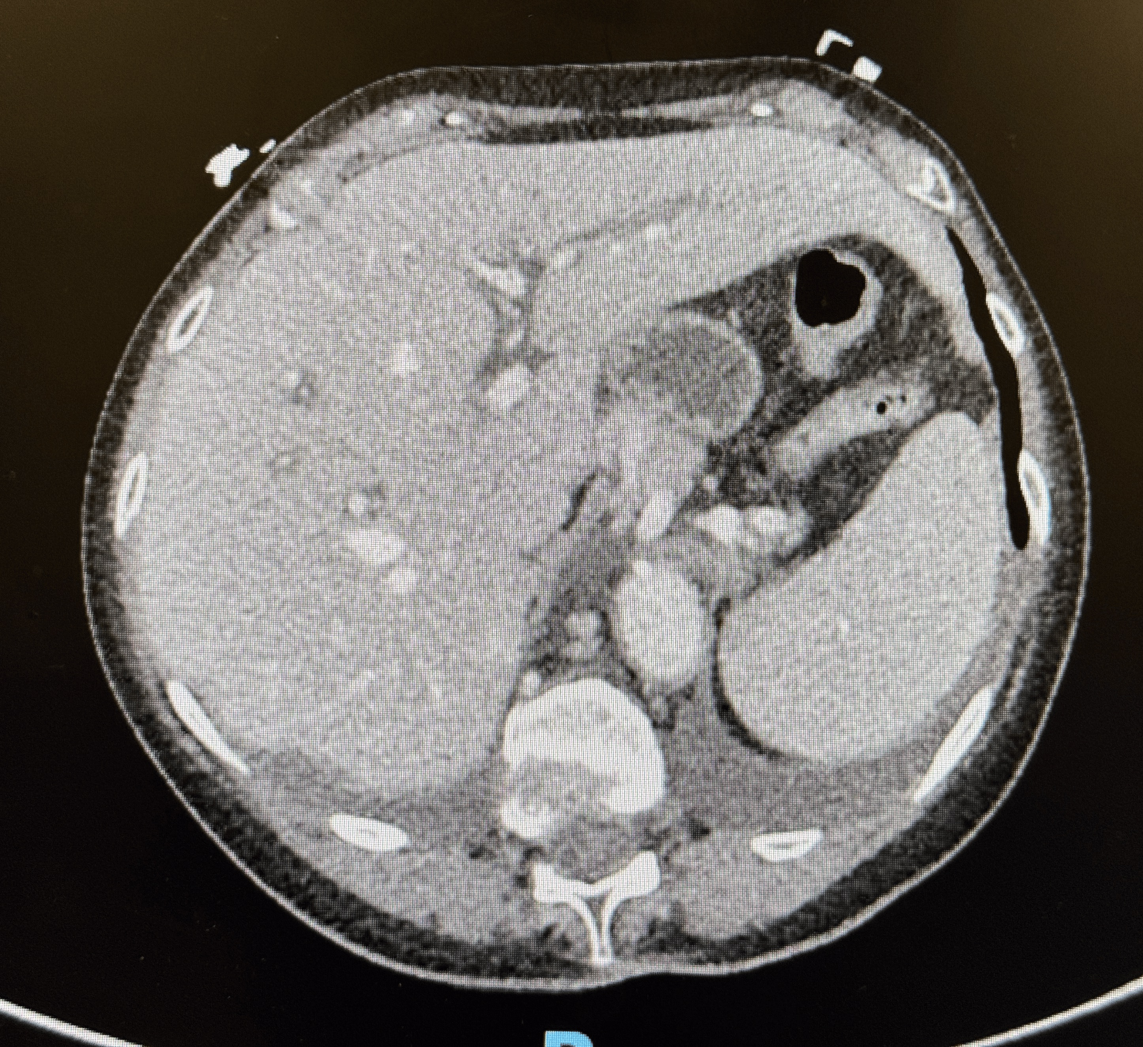
Abdominal CT showing hepatosplenomegaly.

Chest CT revealed extensive ground-glass opacities with interlobular septal thickening, while abdominal CT confirmed hepatosplenomegaly (Figures 2, 3).

**Figure 4:**
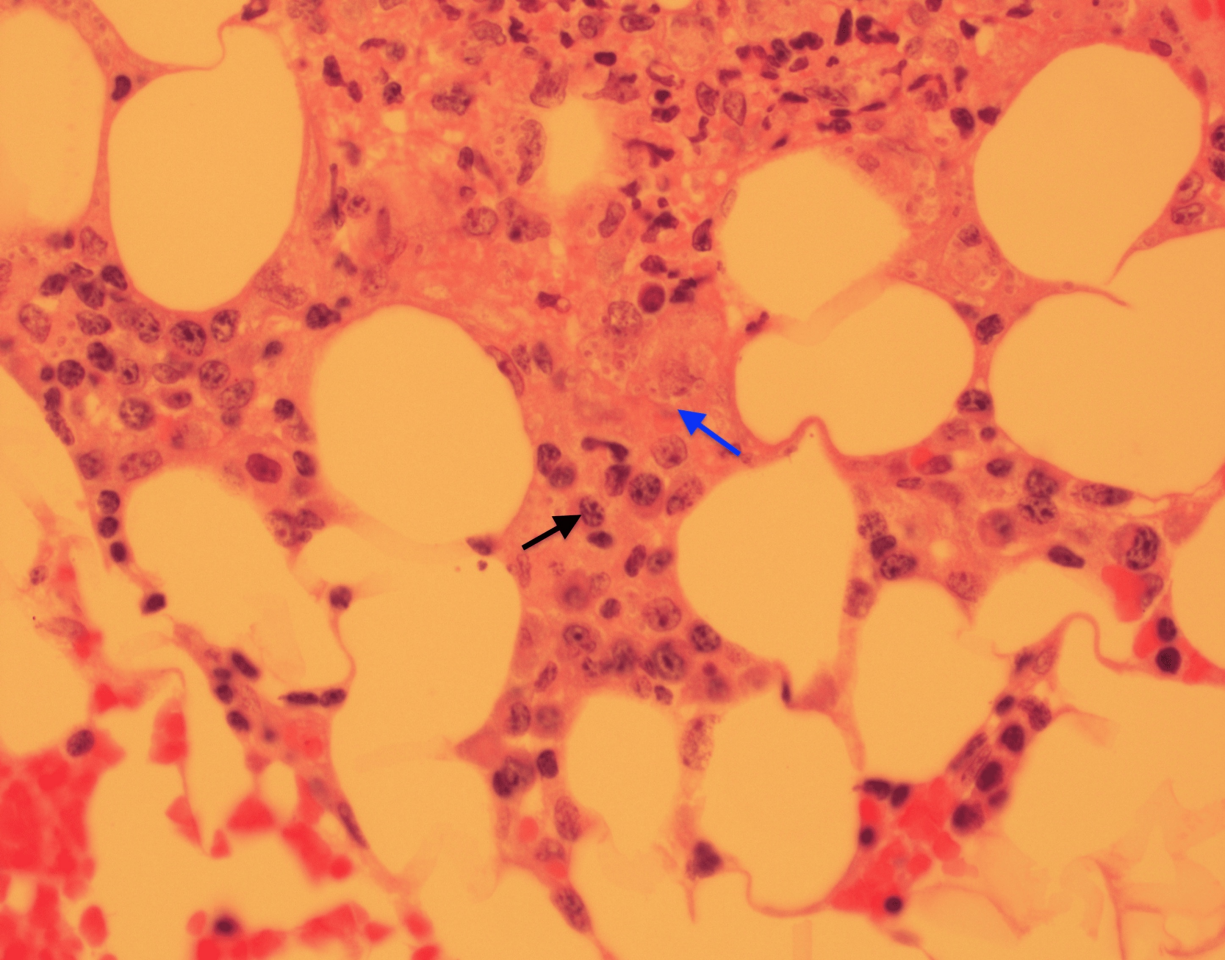
Bone marrow biopsy demonstrating granulomatous inflammation with intracellular yeast forms consistent with Histoplasma capsulatum (black arrow), occasional macrophages, decreased trilineage hematopoiesis, and granuloma (blue arrow).

Bone marrow biopsy demonstrated granulomatous inflammation with intracellular yeast forms consistent with *Histoplasma capsulatum*, occasional macrophages, decreased trilineage hematopoiesis, and granuloma (Figure 4).

**Figure 5:**
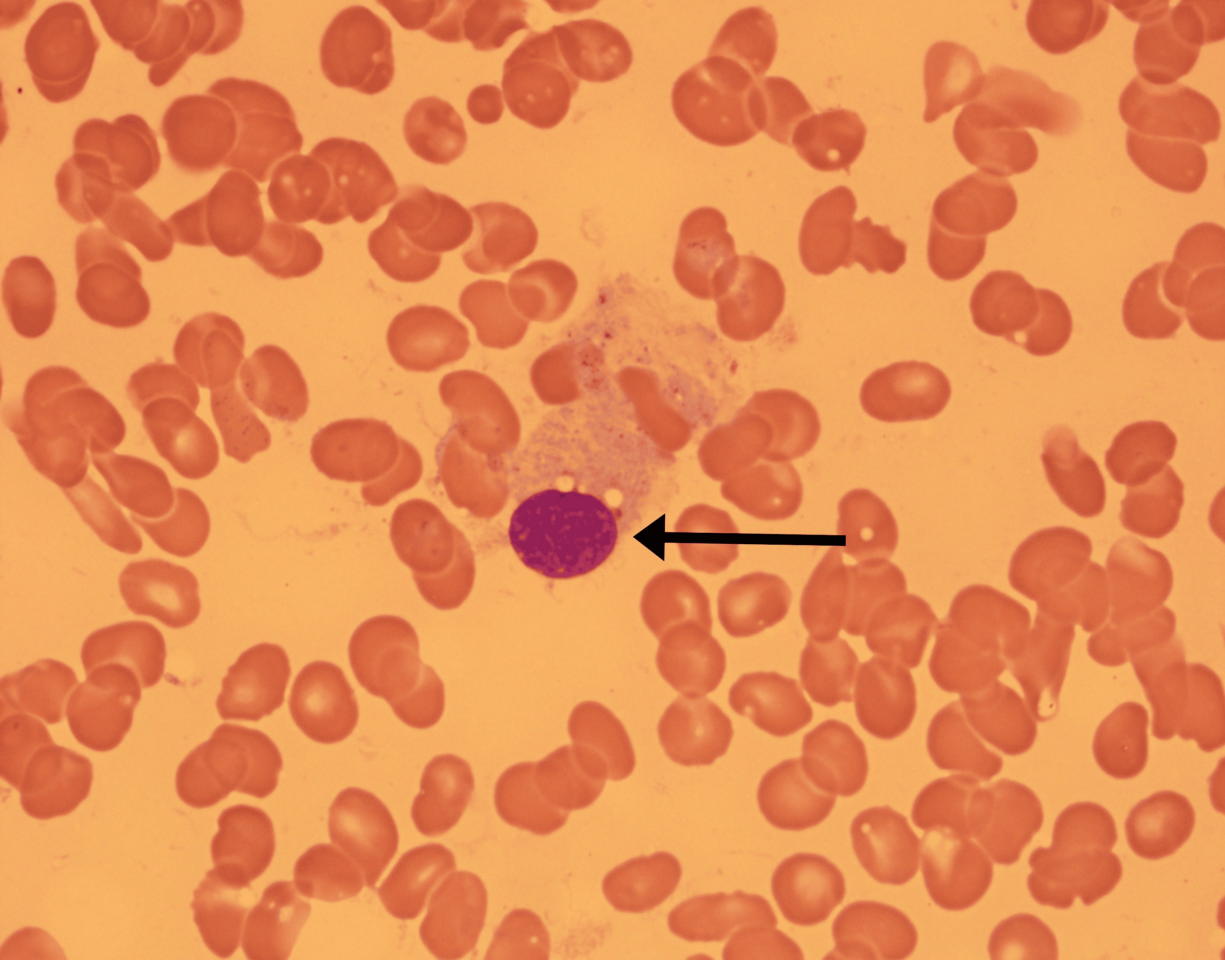
Bone marrow aspirate showing hemophagocytosis of red blood cells by macrophages/histiocytes (black arrow).

Bone marrow aspirate showed hemophagocytosis (Figure 5). These findings confirmed disseminated histoplasmosis as the likely trigger of secondary HLH.

**Figure 6:**
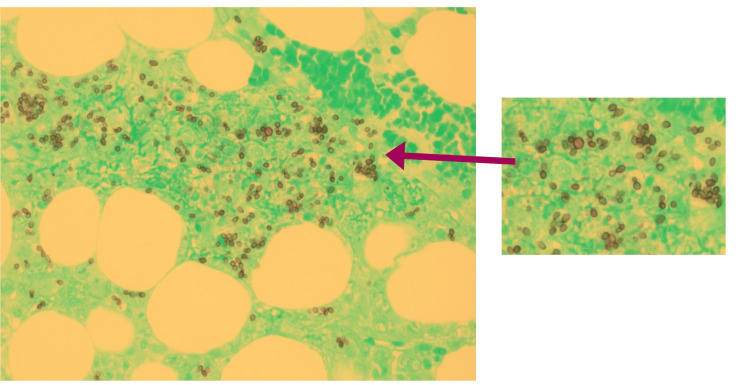
Bone marrow specimen showing Histoplasmosis on Grocott’s methenamine silver stain.

Flow cytometry and fluorescence in situ hybridization analysis of the peripheral blood revealed no monoclonal B-cell population, atypical T cells, or chromosomal abnormalities, effectively ruling out an underlying hematologic malignancy. However, the bone marrow specimen demonstrated the presence of *Histoplasma* organisms on Grocott’s methenamine silver stain (Figure 6). Autoimmune causes were also evaluated, but neither clinical findings nor laboratory results supported diagnoses such as systemic lupus erythematosus, adult-onset Still’s disease, or other autoimmune conditions.

The patient was initially treated with empiric broad-spectrum antibiotics, including cefepime and vancomycin. After the bone marrow biopsy confirmed disseminated histoplasmosis, therapy was switched to amphotericin B for 14 days, followed by itraconazole to complete a one-year course. Filgrastim was administered to address severe neutropenia and lower the risk of secondary infections. Over time, the patient’s condition steadily improved. She continues regular follow-up with the Infectious Disease Clinic and will be finishing her one-year therapy soon. This case highlights the importance of early diagnosis and appropriate treatment of HLH caused by opportunistic fungal infections.

## Discussion

This report describes a case of secondary HLH in an elderly woman with longstanding pancytopenia, precipitated by disseminated histoplasmosis. HLH is an uncommon but highly lethal hyperinflammatory disorder that may develop in association with malignancy, infection, or autoimmune disease. Although viral pathogens are the most frequently recognized triggers, opportunistic fungal organisms such as *Histoplasma capsulatum* can also play a significant role, particularly in older or immunocompromised individuals [1-3]. Our patient satisfied multiple HLH-2004 diagnostic criteria, including unremitting fever, hepatosplenomegaly, cytopenias involving several blood cell lines, striking hyperferritinemia, elevated soluble IL-2 receptor (CD25), and hemophagocytosis on bone marrow biopsy. A ferritin threshold of 500 µg/L is generally considered supportive for HLH, and, in this case, levels were well beyond that value, reinforcing the diagnosis. Similarly, the marked rise in soluble IL-2 receptor reflected heightened immune activation. Bone marrow findings of macrophages actively engulfing hematopoietic cells further substantiated the diagnosis [2-5]. Overall, the patient’s clinical and laboratory features left little doubt about the underlying disorder. HLH is essentially a cytokine-driven hyperinflammatory state capable of progressing quickly to multi-organ dysfunction. Because its presentation often overlaps with severe sepsis, recognition is frequently delayed. Although histological demonstration of hemophagocytosis in the marrow can support the diagnosis, clinical context and laboratory parameters remain central to case confirmation [5-7]. When HLH arises in the setting of disseminated infection, outcomes depend heavily on early recognition, timely antifungal therapy, and aggressive supportive measures, all of which are crucial in reducing mortality [8].

## Conclusions

In elderly patients with chronic pancytopenia who present with fever, low blood counts, and enlarged liver or spleen, HLH should be considered, especially when opportunistic infections such as disseminated histoplasmosis are present. Prompt bone marrow examination, along with assessment of ferritin and soluble IL-2 receptor levels, is essential for early diagnosis and effective management.
